# Efficient Algorithms for Probing the RNA Mutation Landscape

**DOI:** 10.1371/journal.pcbi.1000124

**Published:** 2008-08-08

**Authors:** Jérôme Waldispühl, Srinivas Devadas, Bonnie Berger, Peter Clote

**Affiliations:** 1 Department of Mathematics, Massachusetts Institute of Technology, Cambridge, Massachusetts, United States of America; 2 Computer Science and Artificial Intelligence Laboratory, Massachusetts Institute of Technology, Cambridge, Massachusetts, United States of America; 3 Department of Biology, Boston College, Chestnut Hill, Massachusetts, United States of America; Duke University, United States of America

## Abstract

The diversity and importance of the role played by RNAs in the regulation and development of the cell are now well-known and well-documented. This broad range of functions is achieved through specific structures that have been (presumably) optimized through evolution. State-of-the-art methods, such as McCaskill's algorithm, use a statistical mechanics framework based on the computation of the partition function over the *canonical ensemble* of all possible secondary structures on a given sequence. Although secondary structure predictions from thermodynamics-based algorithms are not as accurate as methods employing comparative genomics, the former methods are the only available tools to investigate novel RNAs, such as the many RNAs of unknown function recently reported by the ENCODE consortium. In this paper, we generalize the McCaskill partition function algorithm to sum over the *grand canonical ensemble* of all secondary structures of all mutants of the given sequence. Specifically, our new program, RNAmutants, simultaneously computes for each integer *k* the minimum free energy structure *MFE*(*k*) and the partition function *Z*(*k*) over all secondary structures of all *k*-point mutants, even allowing the user to specify certain positions required not to mutate and certain positions required to base-pair or remain unpaired. This technically important extension allows us to study the resilience of an RNA molecule to pointwise mutations. By computing the *mutation profile* of a sequence, a novel graphical representation of the mutational tendency of nucleotide positions, we analyze the deleterious nature of mutating specific nucleotide positions or groups of positions. We have successfully applied RNAmutants to investigate deleterious mutations (mutations that radically modify the secondary structure) in the Hepatitis C virus *cis*-acting replication element and to evaluate the evolutionary pressure applied on different regions of the HIV *trans*-activation response element. In particular, we show qualitative agreement between published Hepatitis C and HIV experimental mutagenesis studies and our analysis of deleterious mutations using RNAmutants. Our work also predicts other deleterious mutations, which could be verified experimentally. Finally, we provide evidence that the 3′ UTR of the GB RNA virus C has been optimized to preserve evolutionarily conserved stem regions from a deleterious effect of pointwise mutations. We hope that there will be long-term potential applications of RNAmutants in de novo RNA design and drug design against RNA viruses. This work also suggests potential applications for large-scale exploration of the RNA sequence-structure network. Binary distributions are available at http://RNAmutants.csail.mit.edu/.

## Author Summary

Evolution is a central concept in biology. This phenomenon can be observed at all levels of the organization of life—from single molecules to multicellular organisms. Here, we focus our attention on the implication of evolution at the level of nucleic acid sequences. In this context, RNA sequences presumably have been optimized by evolution to achieve specific functions. These functions are supported by a structure that can be determined using thermodynamics-based models and energy minimization techniques. In this work, we develop efficient algorithms for predicting energetically favorable mutations and study their impact on the stability of the structure. We use these techniques to reveal sequences under evolutionary pressure and design new methods to predict lethal mutations. Applications of our tool lead to a better understanding of the mutational process of some key regulatory elements of two important pathogenic RNA viruses—human immunodeficiency virus and hepatitis C virus.

## Introduction

RNA's ubiquitous role in regulation and development is now understood to be much more important than previously believed. Apart from messenger RNA (mRNA), transfer RNA (tRNA) and ribosomal RNA (rRNA), there are many important enzymatic and regulatory functions of RNA, and it seems clear that we are far from having discovered all *non-coding* RNA (ncRNA) genes (Non-coding RNA [1],[2] is functional RNA that is transcribed, yet does not code for a protein). Indeed, according to the ENCODE Consortium [3], RNA is “pervasively expressed” in the human genome, with approximately 15% of genomic DNA being transcribed, much of it into RNA of no known function.

The functional diversity of non-coding RNA is enormous, ranging from translating mRNA into proteins via the genetic code (tRNA), to catalyzing the peptidyltransferase reaction in appending an amino acid to the growing peptide (rRNA [4]), to directing the chemical modifications of specific ribosomal nucleotides (snoRNA [5]), to the down-regulation of protein product (miRNA [6]), to gene up- or down-regulation by transcriptional and translational modification (riboswitches [7]), to the regulation of alternative splicing ([8]). To achieve their function, non-coding RNAs (except for small RNAs such as miRNA) require a structure well suited to their role. If we assume that ncRNA sequences have been adapted, or optimized, by evolution to fulfill a specific function, it is natural to believe that their structures have been also optimized or at least conserved. This observation is the basis for a family of methods for secondary structure determination using multiple sequence alignment and comparative sequence analysis [9]–[12].

RNA is also a molecule governed by fundamental physical laws, and thus folds according to thermodynamic and kinetic principles. Algorithms using experimentally derived free energy parameters [13] for secondary structure prediction have been successfully designed, implemented and applied in mfold [14] and RNAfold [15]. As a consequence, a series of methods combining thermodynamic principles with evolutionary information [16]–[19] has appeared in the last few years.

RNA molecules are not static, forever frozen in a native structure, but rather transition from one low energy structure to another (slightly different) low energy structure, due to thermal fluctuations. In his seminal work, J.S. McCaskill [20] introduced an algorithm to compute the partition function over all secondary structures as well as the base pairing probabilities. This approach has been significantly extended by Ding and Lawrence [21], who sampled secondary structures from the low energy ensemble.

There is a growing interest in understanding which nucleotides of structurally important ncRNA are inessential, and may be modified with no phenotypic change, and which nucleotides play a critical role in structure, hence function. Indeed, mutagenesis studies are a popular technique for investigating the structure and function of both RNA and protein. In silico exploration of deleterious mutations in RNA secondary structure have thus far been carried out by exhaustive studies, where an available tool, such as mfold [22], Vienna RNA Package [23], or Sfold [24], etc. is applied successively to each 1-point mutant, then to each 2-point mutant, etc. depending on sequence length and available computational time; see, for instance Barash [25]. Clearly, this exhaustive technique cannot be used to study the effect of many pointwise mutations in a large sequence. In contrast, the current paper describes an efficient algorithm, RNAmutants, to investigate the minimum free energy structure *MFE_k_* and Boltzmann low energy ensemble *ε_k_* of all secondary structures of all *k*-point mutants, for each value of *k*. In addition to detecting deleterious mutations, RNAmutants could lead to a better understanding of fast-mutating RNA viruses. By understanding fundamental properties of functional RNAs and their robustness to mutation, there may be ultimate applications of our work to the areas of RNA gene discovery and RNA drug design.

In this paper, we describe a new thermodynamics-based method for the investigation of the mutational secondary structure landscape of a given RNA sequence. State-of-the-art thermodynamics-based, single-molecule methods such as McCaskill's algorithm, use a statistical mechanics framework based on the computation of the partition function over the *canonical ensemble* of all possible secondary structures on a given sequence. Unfortunately, methods such as Zuker's algorithm for minimum free energy structure [14], McCaskill's algorithm for the partition function [20], and the sampling method of Ding and Lawrence [24], do not permit any modification of the input sequence during their execution and thus cannot investigate the mutation landscape of a sequence, except by exhaustive enumeration of all mutated sequences. Indeed, the highly original work on *neutral networks* due to Peter Schuster and the Vienna group [26]–[28] reposes on such experiments where RNAfold is applied to all 4*^n^* many RNA sequences of length *n*. The theory of neutral network is still an active area of research—see the recent review of Cowperthwaite and Meyers [29]. It follows that RNAmutants could be useful for further studies of these networks.

Consequently, except for small exhaustive enumeration studies, such as in the work of Barash [25], no group has been able to answer questions like the following. *What is energetically the most favorable secondary structure adopted by an arbitrary k-point mutant, possibly subject to preserving the location of specific binding sites and possibly constrained by requiring certain positions to be paired resp. unpaired? If an RNA molecule is under evolutionary pressure to adopt a low energy structure, subject to certain constraints (binding site, catalytic core), then which positions are most likely to be mutated and what is the consensus sequence and secondary structure of the low energy ensemble.*

There may be objections to what may seem to be yet another thermodynamics-based RNA structure algorithm that we present in this paper, since it is known that RNA secondary structure prediction algorithms that incorporate comparative genomics (multiple structural alignments) generally predict structure more accurately than do single-molecule, thermodynamics-based algorithms such as mfold, RNAfold, and Sfold. See work of Gardner and Giegerich [30], who show for instance the more accurate performance of Pfold, a program of Knudsen and Hein [18] that depends on an explicit evolutionary model and a probabilistic model for structures.

There are two answers to this objection. First, our program RNAmutants performs computations and admits biological applications that no other software can realize, regardless of whether the software is based thermodynamics or comparative genomics. Second, recent findings of the encode project consortium [3] indicate that the human genome is “pervasively expressed,” with many RNA transcripts of unknown function having no homology to known RNA families. While comparative genomics has successfully been used to investigate the structure and evolution of RNAs for which reliable multiple alignments exist, only thermodynamics-based methods can be applied to novel RNAs, such as those reported by the encode consortium. Given the existence of highly reliable, multiple structural alignments of RNAs of the same class, it makes sense to apply comparative genomics methods, such as Pfold of Knudsen and Hein [18], the phylogenetic stochastic context-free grammar (phylo-SCFG) program EvoFold of Pedersen et al. [11], or the Bayesian MCMC program SimulFold of Meyer and Miklós [12]. In the absence of highly reliable multiple alignments, such as with the raw data of the encode consortium, thermodynamics-based algorithms are not just the only alternative, but such algorithms in general perform rather well. Indeed, on average, the predicted MFE structure contains 73% of known base-pairs when tested on domains of fewer than 700 nt; cf. Mathews et al. [31].

In previous work [32], we introduced a novel formal grammar framework (AMSAG) to compute the *δ*-superoptimal structure. By *δ*-superoptimal structure, we mean the minimum free energy (MFE) structure among all sequences *ω′* with a string edit cost of at most *δ* from the input sequence *ω* (i.e., *ω′* such that *d*(*ω*,*ω′*)≤*δ* for a given edit distance *d*). Hence, in principle AMSAG can handle any edit operation (e.g., mutation, insertion, and deletion). However, in addition to the difficulty of estimating good edit costs, the time required to compute the *δ*-superoptimal structures can be prohibitive, even for small values of *δ*.

To overcome these problems, in subsequent work [33], we refined the problem by restricting our sequence search space to *k*-mutants (i.e., sequences differing of exactly *k* mutations with the input sequence). This simplification allowed us to design and implement an efficient algorithm to compute the partition function over all secondary structures of all *k* -point mutants, with respect to the Nussinov energy model [34]. (The Nussinov energy model ascribes an energy of −1 per base pair, while ignoring any destabilization due to loops. In contrast, the Turner energy model [13] ascribes experimentally measured, context-dependent free energies for base stacking, as well as positive, destabilizing free energies for various types of loops: hairpins, bulges, internal loops and multiloops. It is well-known that the Nussinov energy model is too simplistic to permit reasonable applications of the kind presented in this paper.)

However, due to AMSAG's generality, it is technically difficult to incorporate the full Turner energy model [13] into the AMSAG framework. In order to circumvent these difficulties, we have designed new multiple recursions, allowing for a technical breakthrough to develop RNAmutants, a unified algorithm to compute the minimum free energy structure *MFE*(*k*) and partition function *Z*(*k*) over all *k*-point mutants of a given RNA sequence, even admitting *constraints* of two forms—sequence identity constraints (certain positions, such as those known to be important for protein binding are not allowed to mutate), and structural constraints (certain positions are required to pair or to be unpaired). RNAmutants uses the state-of-the-art Turner energy model [13] *without* dangles. (A dangle is a single-stranded nucleotide, occurring either 5′ or 3′ to a base pair. This energy model corresponds to RNAfold -d 0 in the Vienna RNA Package. In a first implementation with dangles, the computational overhead caused by including dangles was so prohibitive that we decided not to implement them in the final version of RNAmutants.)

Using our partition function, we explore the mutation landscape of a given RNA sequence by sampling not from the uniform distribution of *k*-point mutants, but rather from the Boltzmann distribution of low energy *k*-point mutants.

**Figure 1:**
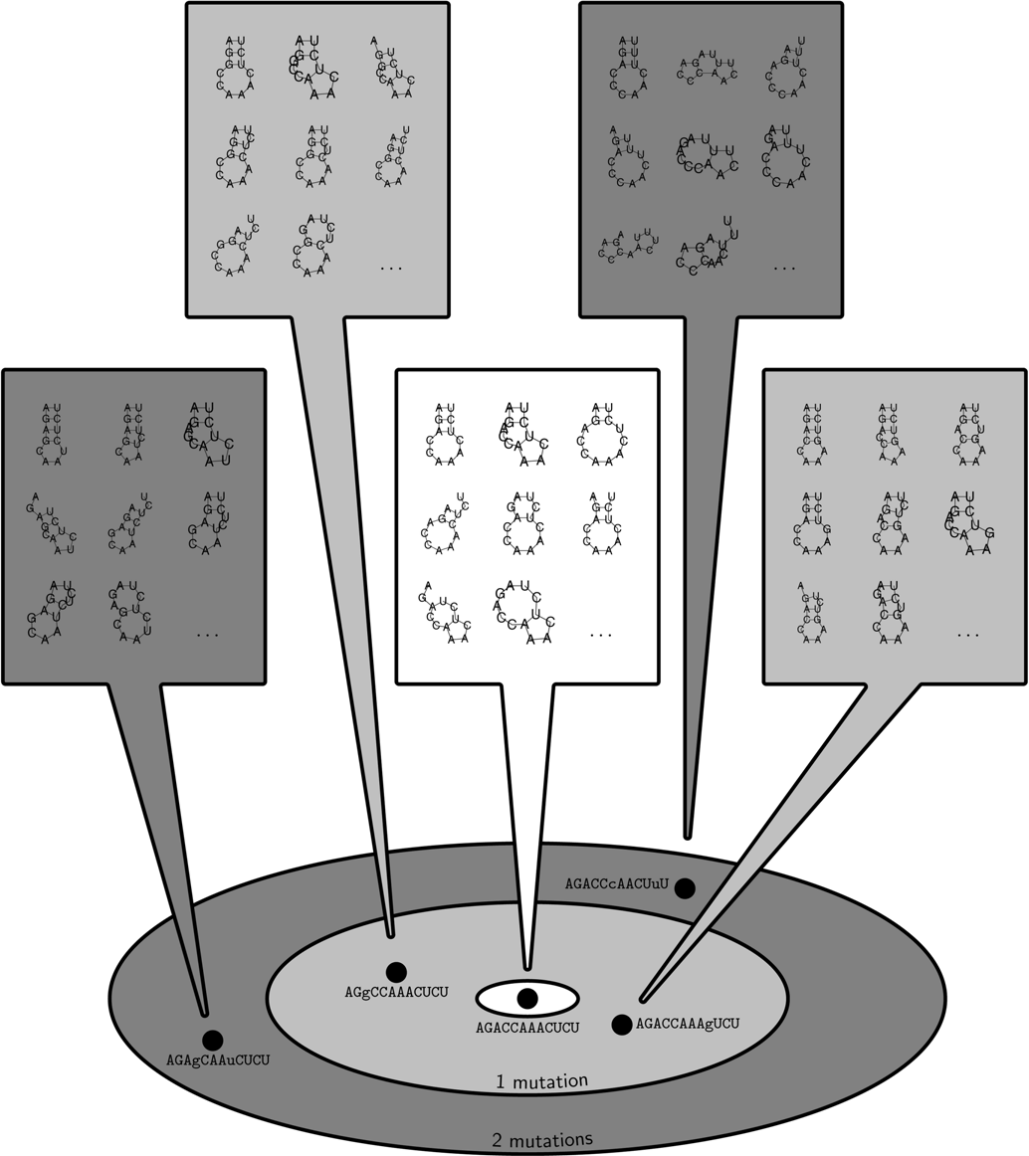
Schematic representation of the *k*-mutant Boltzmann ensemble sampled by RNAmutants. The input RNA sequence is represented at the center while the *k*-neighbourhoods (Here *k*  =  1, 2) are represented by concentric rings. Each individual RNA sequence is associated with a set of secondary structures that can be mapped onto it (the boxed structures). These comprise the set of structure that have to be enumerated to compute the Boltzmann partition function).

RNAmutants naturally extends the classical RNA secondary structure model. Instead of considering the set of secondary structures that can be built on the input sequence alone, as do mfold, RNAfold, and Sfold, we consider all secondary structures of all sequences with at most *k* mutations. In other words, given an RNA sequence of length *n* and an integer *k*_max_≤*n*, we compute the partition function *Z_k_* over all secondary structures of all *k*-point mutants, for all *0*≤*k*≤*k*_max_. When *k* = 0, we obtain McCaskill's partition function. The approach is illustrated in Figure 1.

We then extend the range of techniques developed in previous work [32],[33] for mutant RNAs and present a sampling algorithm allowing us to sample mutant sequences, together with their sampled secondary structure, from the low energy ensemble. A novelty of our algorithm is to sample mutations according to their weight in the Boltzmann ensemble. This result generalizes the RNA secondary structure sampling algorithm of Ding and Lawrence [21]. From sampling, we derive a novel method to predict mutations disrupting the secondary structure of the original sequence (a.k.a. deleterious mutations).

Here, we provide a technical breakthrough far beyond brute force computational techniques in the work of Barash [25] and of Shu et al. [35]. Since there are 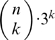, or roughly *n^k^*, many *k*-point mutants of an RNA sequence of length *n*, any method relying on exhaustive listing of all *k*-point mutants has only a limited range of applicability.

We tested our algorithms on six different families of RNA sequences from Hepatitis C and Human Immunodeficiency viruses available in the Rfam database [9], as well as the 3′ UTR of GB virus C. We then compared our results with experimental studies [36]–[39], to investigate the robustness of RNA structures and the nature of deleterious mutations. We performed five types of computational experiments, thus showing the range of possibilities afforded by RNAmutants. First, we demonstrate the computational efficiency of RNAmutants by computing the partition function over all possible mutants (i.e., all *k*-mutants, for *0*≤*k*≤*n*, where *n* is sequence length), and by sampling we estimate the probability of mutation of each nucleotide of the given sequence. Second, we analyze the robustness of RNA structures to point-wise mutations of the wild-type sequence, over a collection of 2806 sequences taken from five different families of RNA elements from hepatitis C virus (HCV) and human immunodeficiency virus (HIV). From our analysis of HCV and HIV, we make some observations concerning possible application to RNA gene discovery and drug design. Third, using previously published experimental results [39], we evaluate the accuracy of our predictions of deleterious mutation predictions for the hepatitis C virus *cis*-acting replication element (HCV CRE). We suggest new possible mutation sites which have not been previously detected or tested. Fourth, we show how our techniques can be used to identify regions that have been constrained during evolution to conserve patterns preserving the (functional) structure of a given RNA. In this fashion we can predict nucleotide sites likely to be under *purifying* selective pressure. Taken altogether, our applications of RNAmutants provide a better identification and understanding of those critical areas of an RNA secondary structure. Finally, by scanning of the 3′ UTR of the GB RNA virus C with a fixed size frame, we show how RNAmutants can be used to perform genome-scale analysis and offer a novel insight inside the genome structure that cannot be achieved through other approaches. More specifically, we provide evidence that the sequence has been optimized to preserve evolutionarily conserved stem regions from a deleterious effect of pointwise mutations.

## Methods

We present in this section the theoretical results achieved in this paper.

### McCaskill's Partition Function

We build our algorithms upon the seminal McCaskill's recursions [20]. Hence, for the benefit of the reader, we give a brief presentation of McCaskill's algorithm.

Given RNA nucleotide sequence *a*_1_,…,*a_n_*, we will use the standard notation 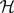 to denote the free energy of a hairpin, 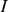 to denote the free energy of an internal loop (combining the cases of stacked base pair, bulge, and proper internal loop), while the free energy for a multiloop containing *N_b_* base pairs and *N_u_* unpaired bases is given by the affine approximation *a*+*b N_b_*+*c N_u_*.

For RNA sequence *a*_1_,…,*a_n_*, for all 1≤*i*≤*j*≤*n*, the McCaskill partition function *Z*(*i*,*j*) is defined by Σ*_S_* e^−*E*(*S*)/*RT*^, where the sum is taken over all secondary structures *S* of *a*[*i*, *j*], *E*(*S*) is the free energy of secondary structure *S*, *R* is the universal gas constant, and *I* is absolute temperature.

Definition 1 (McCaskill's partition function)

- *Z*(*i*, *j*): *partition function over all secondary structures of a*[*i*, *j*].
- *Z^B^* (*i*, *j*): *partition function over all secondary structures of a*[*i*, *j*], *which contain the base pair* (*i*, *j*).
- *Z^M^* (*i*, *j*): *partition function over all secondary structures of a*[*i*, *j*], *subject to the constraint that a*[*i*, *j*] *is part of a multiloop and has at least one component*.
- *Z^M1^* (*i*, *j*): *partition function over all secondary structures of a*[*i*, *j*], *subject to the constraint that a*[*i*, *j*] *is part of a multiloop and has at exactly one component. Moreover*, *it is required that i base-pair in the interval* [*i*, *j*]; *i.e.*, (*i*, *r*) *is a base pair, for some i*<*r*≤*j*.

Before continuing, we remark here that in our implementation of McCaskill's algorithm and its far-reaching extension, RNAmutants, we parse the free energy parameters from tables of mfold 2.3 for all temperatures 0 to 100 in degrees Celsius (for reliable free energies at temperatures other than 37 °C). RNAmutants also allows the user to choose to apply the newer mfold 3.0 energy parameters at 37 °C. Affine parameters *a*, *b*, and *c* for multiloops are taken from mfold tables as well.

**Figure 2:**
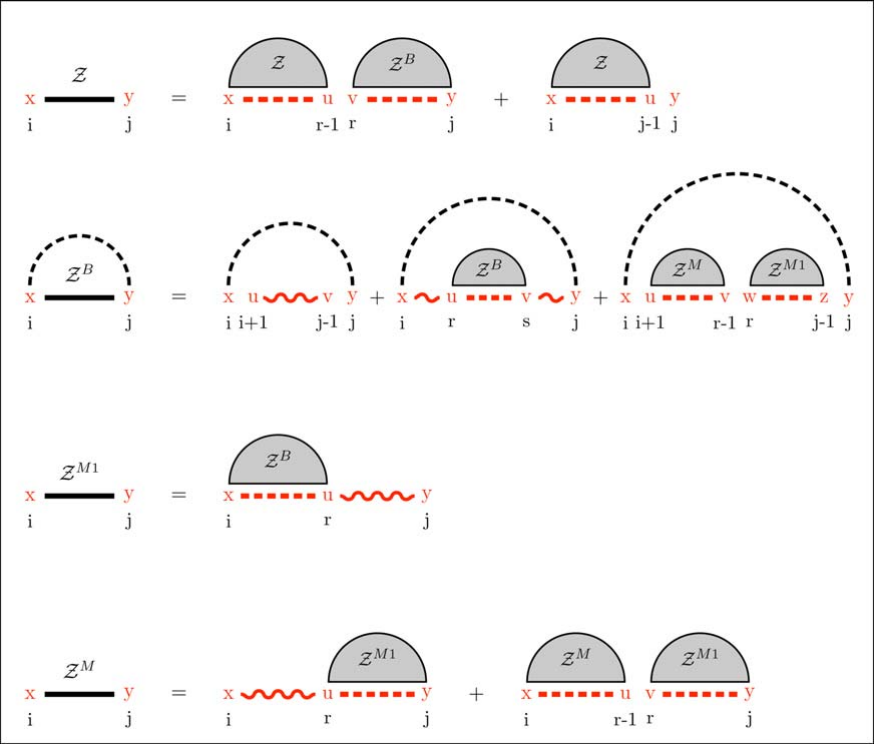
Feynman diagram of original recursions from McCaskill's algorithm [20] to compute the partition function and extension (in red) to RNAmutants recursions. Sequence index are given below the diagram. Shaded half-disks represent secondary structures with at least one base pair and correspond to recursive calls of the partition function computations. The labels give the type of the recursion. The dashed arc lines represent base pairs. The extensions brought by RNAmutants to the McCaskill recursions are highlighted in red and address the labeling of the mutant sequence. The distribution of the mutations is determined using the recursive equations described in the section Partition Function for Mutant RNA in Methods. Wavy lines represent ensembles of sequences with a fixed number of mutations and an empty secondary structure. While dashed lines are mutant sequences to be recursively determined.

With this, we have the unconstrained partition function
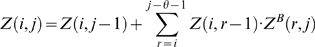
(1)The constrained partition function closed by base pair (*i*, *j*) is given by
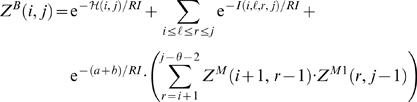
(2)The multiloop partition function with a single component and where position *ii* is required to base-pair in the interval [*i*, *j*] is given by
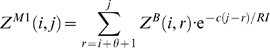
(3)Finally, the multiloop partition function with one or more components, having no requirement that position *i* base-pair in the interval [*i*, *j*] is given by
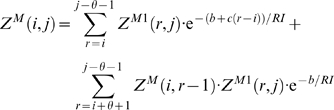
(4)See Figure 2 for a pictorial representation of the recursions of McCaskill's (original) algorithm [20]; note that the recursions are are not quite the same as those given in [15].

### Partition Function for Mutant RNA

We now turn to our mutational partition function and show how to generalize the original McCaskill's recursions.

In the following, a base pair between nucleotide *a_i_* and *a_j_* is denoted by the ordered pair (*i*, *j*). When we wish to consider the nucleotides of this base pair, we write 〈*x*, *y*〉, where *x* = *a_i_*, *y* = *a_j_*. In short, round brackets connote nucleotide positions, while angle brackets connote nucleotides.

Since we consider mutations, we need to introduce energy parameters for hairpins, stacked base pairs, bulges, and internal loops, in which nucleotides and sometimes their neighboring nucleotides are explicitly given. Parameters for multiloops remain unchanged. This is done in the following definition.

Definition 2 (Generalized free energy parameters)

*Let x*,*x′*,*y*,*y′*,*u*,*u′*,*v*,*v′ denote nucleotides, and ℓ*, *ℓ_1_*, *ℓ_2_ denote lengths*.

- *hairpin*(*x*,*u*,*v*,*y*, *ℓ*): *Free energy of a hairpin closed with the base pair* 〈*x*, *y*〉 *and with the nucleotides u and v at the leftmost and rightmost extremities of the loop of size ℓ*.
- *stack*(*x*,*u*,*v*,*y*): *Base stacking free energy when the base pair* 〈*x*, *y*〉 *stacks on the base pair* 〈*u*, *v*〉.
- *bulge*(*x*,*u*,*v*,*y*, *ℓ*): *Free energy of a bulge closed between base pairs* 〈*x*, *y*〉 *and* 〈*u*, *v*〉 *and having ℓ nucleotides in the bulge*.
- *internal*(*x*,*x′*,*u′*,*u*,*v*,*v′*,*y′*,*y*, *ℓ_1_*, *ℓ_2_*): *Free energy of an internal loop closed between base pairs* 〈*x*, *y*〉, 〈*u*, *v*〉, *where x′*, *y′* (*resp. u′*, *v′*) *are the immediate neighbors of* (*x*, *y*) (*resp. u*, *v*) *within the loop*. *The length of the left* (*resp. right*) *loop is ℓ_1_* (*resp. ℓ_2_*).

Free energy parameters used in the functions in Definition 2 come from the most current nearest-neighbor model described in [13].

Our recursions require the following notation. Let 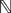 denote the set of RNA nucleotides A, C, G, and U, and let 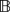 denote the set of Watson–Crick and wobble pairs AU, UA, GC, CG, GU, and UG. The number of *k*-point mutants of a given RNA sequence of length *n* is clearly equal to
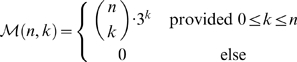
We use the Kronecker delta function, defined by
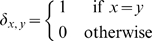
As well, let *σ_x,y_* = 1−*δ_x,y_*. As we will see in the following, these notations allow to keep the structure of the McCaskill algorithm [20] unchanged and thus generalize its principle. In consequence, we use the same partition function arrays given in definition 1, but extend them to keep track of the number of mutations *k* and the nucleotides *x* and *y* at the extremities of the sequence (i.e., at index *i* and *j*). In other words, we add the fields *k*, *x*, and *y* to the partition function arrays.

We now begin the recursion equations. Given RNA sequence *a*_1_,…,*a_n_*, the *k*-point mutant partition function for interval [*i*, *j*] with nucleotide *x* at position *i* (*a_i_*  =  *x*) and nucleotide *y* at position *j* (*a_j_*  =  *y*) is given by
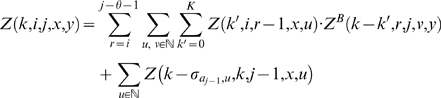
(5)where 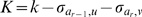. In the sequel, we show how to compute *Z^B^*.

To compute *Z^B^*, we need first to compute the partition functions for hairpins 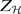, for stacked base pairs 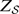, for bulges 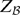, for internal loops 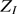, for multiloops of exactly one component, and form multiloops of at least one component.

The partition function for a hairpin is given by
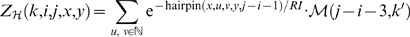
(6)where 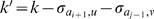. The partition function for a stacked base pair is given by
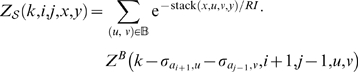
(7)The partition function for a bulge is computed by summing over all possible opening base pairs 〈*u*, *v*〉∈B at one extremity of the bulge, over all bulge sizes *b*, and over the number *m* of mutations in the bulge. The location of the bulge (left or right) must be distinguished. To simplify the notations we let Δ denote *j*−*i*−*3*−*θ*.
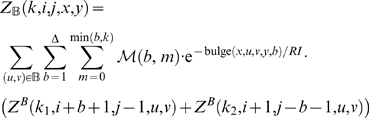
(8)where 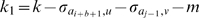 and 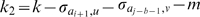. The recursion associated with an internal loop is an extension of that for bulges. We sum over all possible base pairs 〈*u*, *v*〉∈B at the extremity of the internal loop and consider all possible nucleotides *x′*, *u′*, *v′*, *y′* adjacent to the base pairs defining the loop. All possible lengths for the left (*ℓ*_1_) and right (*ℓ*_2_) portions of the internal loop are considered, and we distribute 0≤*m*≤min(*ℓ*_1_ + *ℓ*_2_,*k*) mutations within the loop, the remaining mutations left for the component closed by (*u*, *v*). Since there are special energy parameters for 1×1, 1×2, 2×1 (and 2×2) internal loops, these cases are treated independently; i.e., when *x′* = *u′* or *v′* = *y′*. For readability, we suppress these latter loop details, although they are handled correctly in the program RNAmutants. Denote Δ′  =  *j*−*i*−7−θ. The partition function for internal loops is given by
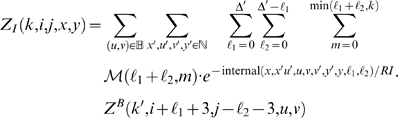
(9)where 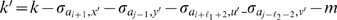.

We now focus on the formation of multiloops, first considering the computation of *Z^M^* ^1^ for multiloops having a single component. The definition of *Z^M^* ^1^(*k*,*i*,*j*,*x*,*y*) requires that position *i* base-pair in the interval [*i*, *j*], so we consider all intermediate positions *i*<*r*≤*j* which might base-pair with *i*, and distribute the required *k* mutations among the component closed by (*i*, *r*) and the unpaired bases in the interval [*r*+1, *j*]. This yields
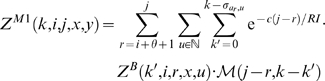
(10)Now we consider the partition function *Z^M^* (*k*,*i*,*j*,*x*,*y*) for multiloops of one or more components, without the requirement that position *i* base-pair. There are two cases to consider. First, we determine an intermediate position *i*≤*r*<*j* for which there is a multiloop with exactly one component closed by base pair (*r*, *s*) for some *r*<*s*≤*j*, and all bases in the intervals [*i*, *r*−1] and [*s*+1, *j*] are unpaired. This case is handled by a recursive call to *Z^M^* ^1^, where we distribute the *k* mutations among the intervals [*i*, *r*−1] and [*r*, *j*]. In the second case, we determine a multiloop of one component closed by a base pair of the form (*r*, *s*) where *i*<*r*<*s*<*j* and recursively consider the multiloop on the interval [*i*, *r*−1]. Again, *k* many mutations must be distributed between the left and right multiloops. This yields the following
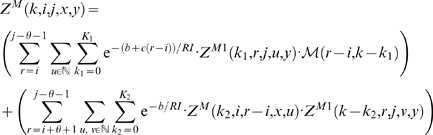
(11)where 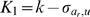 and 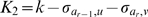.

We can now formalize the recursion for the constrained partition function *Z^B^*(*k*,*i*,*j*,*x*,*y*) closed by base pair (*i*,*j*). This function is defined by
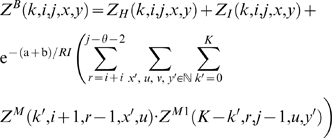
(12)where 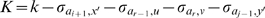.

For a given RNA sequence of length *n*, we define the partition function for *k*-point mutants by
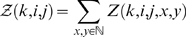
Finally, given a length *n* RNA sequence, the (complete) partition function for mutants is given by
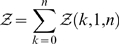

Figure 2 illustrates these recursive equations using Feynman diagrams. Drawing on analogous notions from thermodynamics, we may consider McCaskill's partition function [20] to be over the *canonical ensemble* of all secondary structures of a given RNA sequence, while the (complete) mutant partition function is over the *grand canonical ensemble* of all secondary structures of all mutants of the given sequence.

### Computational Complexity

The computation of the complete partition partition of the grand canonical ensemble of a sequence of length *n* is achieved in time *O*(*n^5^*) and space *O*(*n^3^*). Compared to the original complexity of the McCaskill partition function algorithm (*O*(*n^3^*) in time and *O*(*n^2^*) in space), the increase of the complexity in space can be imputed to the necessity to add a parameter in the dynamic array to memorize the exact number of mutations occurring between two index *i* and *j*. While the increase in the time complexity results from the enumeration of all configurations obtained from the concatenation of these two arrays in Equation 11.

In practice the enumeration of the eight index at the extremities of the internal loops in Equation 9 generates a large constant overweighting this recursion. The growth of the weight of this phenomena in the time complexity saturates once more than eight mutations are performed since no more mutation can be performed in the configuration. However, the constant remains large and for usual RNA sequence lengths (few hundreds) the time complexity may be dominated by this term.

**Figure 3:**
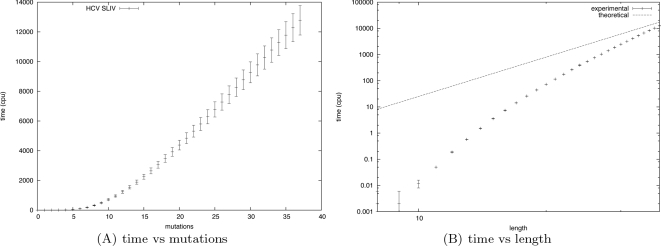
Time complexity measured for all Hepatitis C virus (HCV) stem-loop IV (SLIV) sequences from the Rfam seed alignment. (A) The *x*-axis represents the maximum number *k* of mutations, while the *y*-axis represents the time (in seconds) required by RNAmutants to compute the partition function *Z_i_* for each 0≤*i*≤*k*, and to sample 10 sequences and structures from the corresponding Boltzmann ensemble. Input length of HCV SLIV sequences is 37 nt. The average time over all 110 seed sequences of HCV SLIV is indicated by tick marks, while error bars represent ±1 standard deviation. (B) The *x*-axis represents the length of the input sequence, while the *y*-axis represents the time (in seconds) required by RNAmutants to compute the complete partition function *Z* for *all* mutants (i.e., all possible sequence of a given length). A logarithmic scale is used for both axis. For each length, the average time over five random sequences is indicated by tick marks, while error bars represent ±1 standard deviation. For comparison, a curve *y* = *K*·*x* ^5^ representing the theoretical bound of the time complexity is also plotted.

Curves illustrating time performances of RNAmutants in function of the number of mutations performed for a fixed size input or of the length of the input sequence are given in Figure 3. Figure 3A shows the time required for each value of *k* for a 37 nucleotide sequence (Hepatitis C virus stem-loop IV). Statistics have been computed for the 110 sequences of the Rfam seed of the Hepatitis C virus stem-loop IV.

Figure 3B shows the time required to compute the complete partition function over *all* mutants of a given length *N*. We computed the statistics over five random sequences of size *0*≤*N*≤37. The experimental complexity progressively converges toward the theoretical bound of *O*(*n^5^*). The gap observed between the two curves for small values of *N* can be explained by (i) the combinatorial explosion of the internal loops configurations detailed above and (ii) the fact that the maximum length of internal loops is not reached. (This upper bound is usually set to 30 and is used to justify a time complexity of *O*(*n^5^*).)

### Sampling RNA *k*-Mutants

The sampling procedure follows the classical stochastic backtracking method introduced by Ding and Lawrence [21]. Complexity improvements using the boustrophedon technique recently introduced by Ponty [40] may also be adapted, but for purposes of clarity, such improvements are not discussed here. (In work of Ding and Lawrence [21], sampling RNA secondary structures, given the McCaskill partition function, has worst-case run time *O*(*n^2^*), where *n* is RNA sequence length. In contrast, Ponty [40] shows how the boustrophedon sampling method requires run time *O*(*n* log *n*) in the worst case. In addition, Ponty proves an average-case run time improvement from *O*(*n* √*n*) to *O*(*n* log *n*).)

The main novelty of our sampling algorithm is that in addition to a sample secondary structure traditionally output by RNA sampling algorithms [21],[40],[41], it also outputs a sample *k*-mutant RNA sequence. Indeed, the algorithm will output of a series of sequences with *k* mutations, together with secondary structures for these sequences.

Once the partition function is computed and the dynamic programming tables are filled, we proceed to a stochastic backtracking using the values stored in the arrays, together with the equations given in the previous section, to (randomly) decide which parameters will be used for each recursive calls.

**Figure 4:**
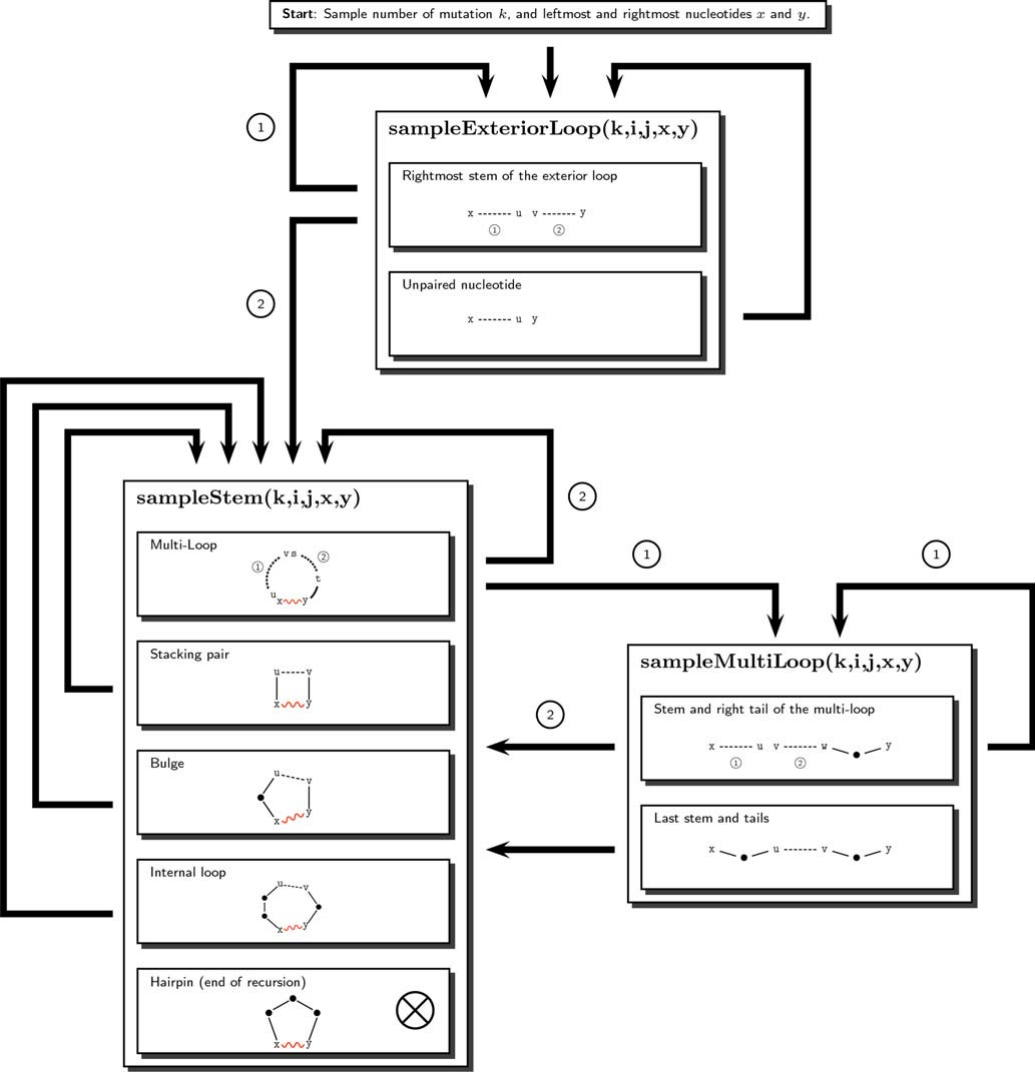
Overview of the sampling procedure. Dashed lines represent the regions which must be recursively sampled. The recursive calls are indicated by an arrow, and labeled when multiple recursive calls are performed. Wavy lines show the base pairs created duringthe execution of the algorithm. Dots indicate nucleotides sampled in the function and are never involved in a recursive call. The number of mutations is determined using the recursive equations of the section Partition Function for Mutant RNA in Methods.

The algorithm uses three functions to sample each basic type of secondary structure motif (e.g., exterior loop, stem and multiloop). An overview of the complete procedure is given in Figure 4. The process starts by randomly choosing the initial parameters *x* (the leftmost nucleotide) and *y* (the rightmost nucleotide) and eventually *k* (number of mutations). In contrast, if desired, for fixed value of *k*, one can sample precisely the Boltzmann weighted *k*-point mutants. The probability of such a configuration is given by 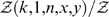. Then, we sample the exterior loop with the function *sampleExteriorLoop* and recursively call the function *sampleStem* to build each type of loop (i.e. hairpin, stacked pair, bulge and internal loop). An exception is for multiloops which use the function *sampleMultiLoop*. The recursions end each time when a hairpin is created inside the function *sampleStem*.

### Using Sampling To Predict Deleterious Mutations

A *deleterious* mutation in RNA is a nucleotide mutation which alters the structure or function of the molecule. For example, the catalytic core of the *Tetrahymena thermophila* group I intron contains a well-defined guanosine binding pocket, whose geometry depends on the secondary and tertiary structure adopted by the intron. Disruption of binding ability caused by a mutation leading to a different structure would be termed deleterious.

The prediction of deleterious mutations has recently emerged as a useful and promising research direction [25],[35]. With the exception of the present paper, all current techniques rely on exhaustively enumerating all possible pointwise mutants, followed by the application of available software such as mfold [22], RNAfold [23], or Sfold [24]. Unlike the approach using RNAmutants, such approaches are limited and cannot be applied to long sequences and/or with more than one or two mutations. Consequently, such traditional approaches could well miss potentially critical mutations or groups of mutations.

Our method is described as follows. Given a wild-type sequence and its *native structure* (by native structure, we mean either the secondary structure inferred from the X-ray crystal, or in the absence of crystal structures, the secondary structure inferred by comparative sequence analysis. Often we take the Rfam consensus structure as the native structure), we use RNAmutants to sample an ensemble of 1000 *k*-point mutant sequences and their structures, for each value of *k*, from 0 to the maximum number of mutation allowed, denoted by *k*_max_. (If not stipulated as part of the input, then *k*_max_ *= n*.) To ensure the pertinence of our approach, we first verify that the centroid secondary structure at level 0 (i.e., no mutation) is close to the native structure. Here, by *centroid* structure, not to be confused with Rfam consensus structure, we mean the secondary structure consisting of those base pairs, whose frequency of occurrence in the sampled set is strictly greater than 0.5. Then, at each level 1≤*k*≤*k*_max_, we probe the samples and extract the sequence and structure such that the base pair associated with the mutation does not belong to the native structure. Alternative experiments or more flexible criteria can be adopted, but the latter seemed to give the best compromise between the number of candidates and the relevance of the structural deterioration.

We measured the deleterious effect of a base pair in the mutant structure, which does not occur in the native structure, by using a value called the *break* number. The break number is computed as the number of base pairs that must be removed from the native structure to prevent the formation of a pseudoknot or base triple, if we force the presence of the base pair created by the mutation. In this fashion we quantify the deleterious effect induced by the newly created base pair. A break number of 0 indicates that the new base pair is compatible with the native structure and does not create any pseudoknot or base triple. In lieu of measuring break number, we could have computed the base pair distance between mutant and native structure; however, two topologically very similar structures can have large base pair distance. For instance, both of the structures

GGGGGGGGACCCCCCCC GGGGGGGGACCCCCCCC

((((((.....)))))) .((((((....))))))

are very similar, and both have free energy of −13.80 kcal/mol, yet their base pair distance is 12. For this reason, we introduce and use break number.

Deleterious mutations extracted from the sample set are ranked according to their deleterious effect, i.e., in decreasing order, sorted by break number. A ranking based on the frequency of occurrence of the mutation would not have been necessarily a wise choice. Indeed, this approach would have highlighted those mutations that lower folding energy, since these would the largest weight in the Boltzmann ensemble. Deleterious mutations that break the native structure do not necessarily improve the MFE in the first steps and hence would appear with a lower frequency in the sample set.

## Results/Discussion

We present here the results of our computational experiments, compare them with previously published experimental results, and discuss their significance.

### Evaluation of the Nucleotide Mutation Propensity and Exploration of the Complete Mutation Landscape

We illustrate in this section the computational efficiency of RNAmutants by exploring the full mutation landscape of a family of RNA sequences (i.e., we compute the partition function 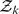 for all 0≤*k*≤*n*). By sampling, we estimate the probability of mutation of each nucleotide by evaluating its effect on the thermodynamic stability of the structure of all *k*-mutants. Additionally, we compute the MFE and the free ensemble energy for all *k*-mutants.

We tested our software on 110 sequences of Hepatitis C virus stem-loop IV (HCV SLIV), each comprising 37 nucleotides, taken from the seed alignment of Rfam [9]. For each sequence, we compute the (complete) partition function over all possible mutants. In the case of the Hepatitis C virus stem loop IV, this represents a total of 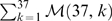 (≈ 1.9×10^13^) sequences. Then, for each sequence and each value of 1≤*k*≤37, we sample 1,000 *k*-point mutants and structures. Per HCV SLIV sequence, this procedure requires about 3 h on a 2.6 GHz AMD 64 byte processor with 250 Mb. The same operation is of course impossible using any classical software such as mfold [14] or RNAfold [15].

**Figure 5:**
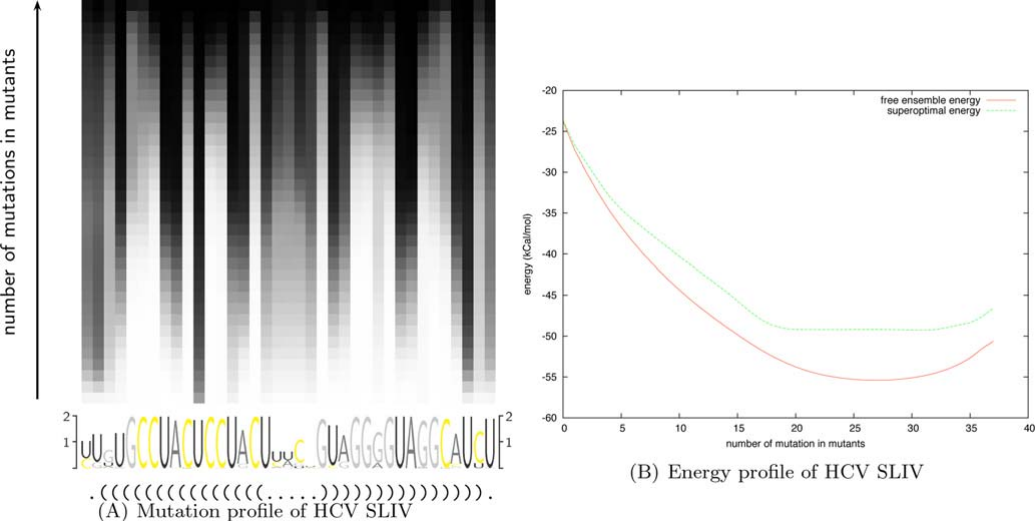
Complete mutation landscape of Hepatitis C virus stem-loop IV (HCV SLIV). (A) Mutation profile of HCV SLIV, averaged over all 110 seed sequences from Rfam, which depicts the probability of mutation of a residue at a level *k* (i.e. among all *k*-point mutants). This profile corresponds to a 37×37 matrix *M* = (*m_x,y_*), where *x* denotes the position within the input HCV SLIV sequence (*x*-axis) and *y* denotes the mutation level or number of mutations (*y*-axis). Mutation frequency computed from sampled structures is represented as a gray level: probability of 1 is depicted as black while probability of 0 is depicted as white, and values of *y* increase from bottom to top. Sequence logo and the consensus secondary structure from the Rfam seed alignment appear below the mutation profile. (B) Superposition of *k*-superoptimal free energy and *k*-mutant ensemble free energy, as computed by RNAmutants; the *x*-axis represents the number of mutations and the *y*-axis represents free energy in kcal/mol. Note that the *k*-mutant ensemble free energy −*RI* ln *Z_k_* is *lower* than the *k*-superoptimal free energy, a situation analogous to the fact that the ensemble free energy −*RI*⋅ln *Z* is lower than the minimum free energy in the output of RNAfold. This may seem paradoxical, unless one realizes that ensemble free energy is not the same as the *mean* free energy *μ* = Σ*_S_ E*(*S*)⋅*exp*(−*E*(*S*)/*RI*)/*Z*, the latter which can be computed by the method of [53] or by the classical statistical mechanics formula 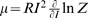 [33].

We show the results in Figure 5. Figure 5A depicts the mutation profile, which gives the probability of mutation of a residue at a level *k* (i.e., among all *k*-point mutants). Here, the profile is displayed as a 37 × 37 matrix with position in the sequence (sequence index) on the *x*-axis and the level *k* on the *y*-axis. The probability of mutation observed over samples is represented as a gray level. A probability of 1 is displayed as a black entry while a probability of 0 is displayed as white. Below the matrix, we also give the sequence logo and the consensus secondary structure from the Rfam seed alignment.

The mutation profile allows us to identify fragile and robust positions in the sequence. In the case of Hepatitis C virus stem-loop IV (HCV SLIV), the secondary structure given by the consensus Rfam seed alignment is a single stem with a tight hairpin loop, without any structural irregularity such as a bulge or internal loop. Such a secondary structure for HCV SLIV is energetically favorable and cannot be drastically improved. Thus, the mutations will tend to conserve the structure and improve the base stacking free energies, while preserving the same base-paired positions. Since the stacking of GC base pairs provides the lowest stacking free energy, all non-GC base pairs will tend to be substituted by GC in the first steps. The sequence logo in Figure 5A confirms this intuition, showing that positions with a clear preference for the nucleotide U, and base-paired with a G in the consensus structure are the first to mutate. Subsequently the nucleotide A tends to be affected, while C and G are relatively well conserved.

All columns present a strictly monotone gradient of color from white to black, thus suggesting that preferred mutation sites are independent and ordered. In addition, the mutation profile shows an alternation of white columns (groups of residues which start to mutate with small value of *k*) and black columns (groups of residues which mutate late). Here, it appears that base-paired positions evolve simultaneously (see, for instance, the motif AU at index 13–14 and UA at index 22–23), presenting examples of compensatory mutations. This phenomenon reveals a stability in the base-pairing of the regions involved, certain to be of interest in RNA design.

In Figure 5B we plot the superposed curves of *k*-superoptimal free energies and *k*-mutant ensemble free energies, as computed by RNAmutants; the *x*-axis represents the number of mutations and the *y*-axis the energy in kcal/mol. Here, *k*-superoptimal free energy is defined as the minimum free energy (MFE) over all mutants having *k* mutations [33], while *k*-mutant ensemble free energy is defined by −*RI log* (*Z_k_*). (In work of Waldispühl et al. [32] and Clote et al. [33], the *k*-superoptimal structure is defined to be the MFE structure over all ≤*k*-point mutants, while in the present paper, it is defined to be the MFE structure over all *k*-point mutants. The current usage seems more appropriate.) These results provide a novel insight into preferential mutation sites as well as structural impacts caused by mutations.

We now analyze the curves of Figure 5B. While the ensemble free energy curve resembles a parabola, the superoptimal free energy curve shows three distinct regions (*k*≤5, then 6≤*k*≤17, and 18≤*k*), each having a linear appearance. Each region reflects the phenomenon described above. From *k*  =  0 to *k*  =  5, the GU base pairs are progressively substituted by GC and the slope is roughly equal to the difference of the stacking free energies associated with both base pairs. Then, the region from *k* = 6 to *k* = 17 is associated with the substitution of AU base pairs by GC, which now requires 2 mutations with a smaller gain of energy. Other optimizations, such as the reordering of nucleotides G and C inside the stem, only bring minor energy improvements and are then performed in the last region (18≤*k*) which presents a flat free energy profile. The characteristic shape of the superoptimal energy curve may be of interest for characterizing sequences that require an optimal secondary structure.

Interestingly, the 5-nucleotide hairpin is very well conserved over sample centroid structures (base pairs with a frequency >0.5 in the sample set—data not shown), even for large values of *k*. Indeed, a tetraloop hairpin might have been expected, due to the energy bonuses assigned to GNRA-tetraloops. This suggests that evolutionary pressure might have designed the sequence to prevent any slippage in the formation of the helix.

Since the secondary structure is conserved throughout the sampled ensemble, the following questions arise. *What function is required by those structural motifs that are preserved in the sampled ensemble? Why did evolution not select a thermodynamically more stable secondary structure in such cases?* Our ability to compute, for the first time, the complete mutation landscape for a given RNA sequence, makes RNAmutants a fundamental tool to address such questions. By using RNAmutants in computational experiments, such as those just described, we can determine putative functionally important motifs and structures that can be subsequently tested experimentally. RNAmutants could lead to important breakthroughs in our understanding of the remarkable combination of robustness and fragility of RNA structures [42].

### Evaluation of the Secondary Structure Robustness Highlights Differences between Families of RNAs in Hepatitis C and HIV Viruses

Estimating how robust a secondary structure is to mutations can be of interest for the characterization of functional RNAs. Here, by sampling structures, we evaluate the conservation of the Rfam consensus structure in the *k*-mutant ensembles, and compare the results obtained from five different families of RNA from Hepatitis C and HIV viruses. These computational experiments highlight major differences between these RNA families and suggest potential application in RNA design.

The method proposed here first samples 1,000 *k*-point mutant sequences and structures for 0≤*k*≤5. To quantify robustness, we compute two notions of distance. First, for each sampled structure *S*, we compute the base pair distance between *S* and the native secondary structure *S*_0_, and thus determine the average over all sampled structures, called *average distance* in the following. Second, we compute the base pair distance between *S* and the sample centroid *S*_c_, where the latter is defined to consist of those base pairs occurring in strictly more than half the sampled structures. (In work of Ding et al. [43], the sample centroid is called the *Boltzmann centroid*, when sampling over all secondary structures using Sfold [24].) This distance is called the centroid distance in the following.

A small average distance means that most sampled structures are identical to the native structure (this entails a small centroid distance as well). A large average distance with a small centroid distance indicates that the core of the native structure is conserved in the sampled structures, while most sampled structures differ from the native structure with respect to a number of base pairs. In this case, the nonnative base pairs in the samples are not well conserved over the ensemble of sampled structures, hence do not appear in the centroid structure. In contrast, a large centroid distance indicates that the same nonnative base pairs are present (or missing) in the majority of sampled structures.

To benchmark robustness, we used (seed) multiple sequence alignments from Rfam [9]. We selected five RNA elements associated with Hepatitis C and human immunodeficiency viruses, each of which is reasonably well predicted by the nearest neighbors energy model, using RNAmutants with 0 mutations, or (equivalently) mfold [22] or RNAfold [23] without dangles. The resulting dataset contains a total of 2,806 sequences. By *native* secondary structure, we mean the Rfam consensus structure from the multiple sequence alignment. Results are given in Table 1.

**Table 1:** Base pair distance between the sampled and native structures for *cis*-regulatory elements from Hepatitis C virus and HIV

RNA	#seq	length	#bp	0	1	2	3	4	5
HCV CRE	52	51.0	14	1.8/0	2.7/0	6.1/2	8.6/1	10.8/9	12.4/10
HCV SLIV	110	35.0	15	0.3/2	0.3/1	0.3/1	0.3/1	0.3/1	0.3/1
HIV PBS	388	94.8	17	12.3/4	14.9/5	16.7/1	17.6/1	18.3/1	-
HIV FE	853	51.9	10	7.6/1	7.7/2	7.7/2	7.6/2	7.4/2	7.2/2
HIV GSL3	1403	81.1	8	9.3/0	9.1/0	8.9/0	9.2/0	9.6/0	-

Native structure is here taken as the Rfam consensus structure from the seed alignments of these elements of HCV and HIV. Two measures are given. The *average distance* represents the average base pair distance between sampled structures and the native secondary structure ***S***_0_. The centroid represents the average base pair distance between sampled structures and the sample centroid ***S***_c_, where the latter is defined to consist of those base pairs occurring in strictly more than half the sampled structures. The number of sequences in the Rfam seed alignment, the average length and the number of basepairs in the native structure are given before the average and centroid distance values.

The structures sampled from the RNA elements of Hepatitis C virus are close to the native structure, while those of human immunodeficiency virus have more base pairs than the native structure. Nevertheless, the centroid structure for samples generated by RNAmutants is reasonably close to the native structure; i.e., centroid distance for RNA elements from HIV is small.

The Hepatitis C virus stem-loop IV (HCV SLIV) is accurately predicted by minimum free energy methods, i.e., Zuker algorithm [14], and despite its small size (35 nucleotides) and large number of base pairs (15), HCV SLIV is also very well conserved in the ensemble of mutants generated by RNAmutants. These results suggest that the RNA nucleotide sequence of HCV SLIV has been thermodynamically optimized and is robust with respect to mutations. In contrast, the secondary structure of sampled mutants of Hepatitis C virus *cis*-acting replication element (HCV CRE) is increasingly divergent as the number of mutations increases. The secondary structure of wild-type HCV CRE sequence is very well predicted by energy minimization methods. The centroid structure of samples generated by RNAmutants, for one to three mutations, changes little and remains very close to the native structure, even if most of the sampled structures have more base pairs than that of the native structure. However, when four or more mutations are allowed, another structure, significantly different from the native one, emerges from the ensemble generated by RNAmutants. This result suggests that HCV CRE has been optimized to resist only a few mutations. This remark suggests the use of RNAmutants to detect those sequences whose structure is locally optimized.

At level 0 (no mutation allowed), in spite of a higher average distance, the centroid structure of the ensemble of samples of HIV RNA elements remains close to the native structure. Interestingly, average distance remains approximately constant when the number of mutations increases. This number even decreases for human immunodeficiency virus primer binding site (HIV PBS). Here again, analysis of mutants generated by RNAmutants seems to confirm the optimization of these sequences to support some functional secondary structure. We note that with similar characteristics (length and number of base pairs) the human immunodeficiency virus frameshit signal (HIV FE) structure appears to be more robust with respect to mutations than is the Hepatitis C virus *cis*-acting replication element (HCV CRE). The centroid structure of human immunodeficiency virus primer binding site (HIV PBS) seems well conserved. In analogy to the phenomenon observed for the Hepatitis C virus *cis*-acting replication element (HIV CRE), the average distance increase suggests that an alternate structure will emerge as the number of mutations increases. Summarizing, we feel that the combination of average and centroid distance measurements is a reasonable tool to estimate the robustness of a structure under mutational variation of a sequence.

Remarkably, the average and centroid distances are very well conserved in human immunodeficiency virus gag stem loop 3 (HIV GSL3), in spite of the huge hairpin loop (69 nucleotides) and very small stem (8 base pairs). One must bear in mind that Rfam consensus structures indicate only those base pairs that are inferred by covariation. It follows that many base pairs may not appear in the centroid structure, such as the 69-nt hairpin. Each of the 8 base pairs in HIV GSL3 is a GC pair, which means that this stem region is not optimized by RNAmutants for the small numbers of mutations *k*. This supports the idea that the mutation robustness of the hairpin loop sequence is optimized.

### Prediction of deleterious mutations in Hepatitis C virus *cis*-acting replication element

In this section, we predict deleterious mutations in Hepatitis C virus *cis*-acting replication element using the method described in section Using sampling to predict deleterious mutations in Methods. We confirm our results by comparing our predictions with previously published experimental results [39]. Moreover, our computational experiments suggest new deleterious mutations which have not been predicted or tested before.

**Figure 6:**
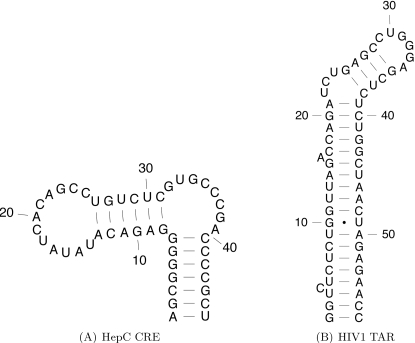
Rfam [9] consensus secondary structure of Hepatitis C *cis*-acting replication element (HCV CRE) and the *trans*-activation response hairpin of the human immunodeficiency virus (HIV1 TAR).

**Figure 7:**
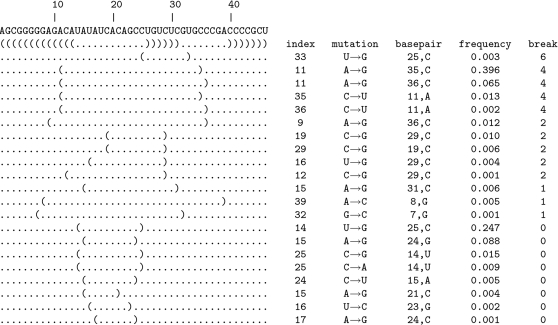
Deleterious mutations identified in the ensemble sampled by RNAmutants on the input of 47 nt Hepatitis C virus *cis*-acting replication element (HCV CRE), known to be essential for viral replication. Pointwise mutants are listed by decreasing order of *break number* (a measure of structural distortion, defined as the number of native base pairs that must be removed for given structure to be compatible with the wild type structure). For each secondary structure listed, we display the base pair associated with the mutation, the mutation type (index and nucleotide substitution), the index and type of the nucleotide that can be associated with the concerned base pair, the frequency of this mutation and the break number.

We performed computational experiments with Hepatitis C virus *cis*-acting replication element (HCV CRE), known to be essential for viral replication. Figure 6 depicts the secondary structure of HCV CRE. To validate our predictive results, we used mutagenesis data from experiments of You et al. [39]. To simplify exposition and enhance clarity of results, we focus our investigation on the prediction of single point deleterious mutations (i.e., *k*_max_ = 1), although of course RNAmutants can be used to infer deleterious noncontiguous groups of mutation sites. Results are given in Figure 7. The top line gives the native secondary structure of the RNA element, while the following lines contain 1-point mutants sampled by RNAmutants. For each pointwise mutant, we display the base pair associated with the mutation, the mutation type (index and nucleotide substitution), the index and the type of the nucleotide that can be associated with the concerned base pair, the frequency of this mutation and the break number.

Here, the HCV CRE sequence has a length of 47 nucleotides, which is slightly shorter than those given in the Rfam multiple alignment. Also, we note a shift of 53 positions between the index of our sequence and those used in [39].

Our results predict the mutation U33G (U86G according to the notation used in [39]) to be the most deleterious. This prediction is confirmed by [39]. In this study, You et al. observed that the mutant C84A/U86G is not viable, while C84A/U86A is still functional. Additionally, it was observed [39] that the mutation U86G is responsible for the alteration of the upper helix (subsequence from nucleotide 8 to 31 in Figure 7) and hence deleterious. However, their results also showed that the single point mutation U86G is still viable, suggesting that this mutation must be supported by C84A to be deleterious. In fact, C84A is suspected to alter the stability of the upper helix, amplifying the ability of U86G to disrupt the structure. The slight overestimation of the deleterious potential of U86G is due to the quality of the energy model used by RNAmutants. Without dangles, the centroid structure is effectively altered by U86G, while with dangles, the mutation C84A is required to disrupt the upper helix (data not shown). The difference is then due to the absence of dangles in the energy model of RNAmutants. However, the deleterious effect of U86G is correctly detected.

The non-viability of other mutants studied in [39] (U71C, C74U, A75U/G76C/C77U, C77U, C90A/A92G, A92G, and C90A) is not attributed to a significant alteration of the native secondary structure. RNAmutants predicts a few other deleterious mutations (with a lower impact)—these are discussed in the following.

The next four deleterious mutations can be grouped in a cluster involving the base pairs (11, 35) and (11, 36). When looking at the 348 sequences in the Rfam seed alignment, it appears that 30 sequences have the mutation C36U, 3 the mutation C35U and 1 the mutation A11G. EMBL accession numbers for the Rfam sequences and the mutations found are shown in Table 2.

**Table 2:** Mutants with mutations A11G, C35U, and C36U in the full alignment of the 47 nt Hepatitis C virus *cis*-acting replication (HCV CRE) element

A11G	
AF054264.1/326-376	(A1G),(A11G)
C35U	
D14853.1/9264-9314	(A15U),(C25G),(G32A),(C35U),(A39G)
D16190.1/986-1036	(A15U),(C25G),(G32A),(C35U),(A39G),(C45U)
D16192.1/986-1036	(G2A),(A15U),(C25G),(C35U),(A39G),(C45U)
C36U	
D87352.1/983-1033	(A1G),(A9G),(A13G),(C25G),(U26C),(U30C),(U33G),(C36U)
D37862.1/983-1033	(A9G),(A13G),(A15U),(C25G),(U26C),(U30C),(U33G),(C36U),(U46A)
D49769.1/983-1033	(A1G),(A9C),(C25G),(U30G),(U33A),(C36U)
D37859.1/983-1033	(A9G),(A17U),(C25G),(U30C),(G32A),(U33G),(C36U),(A39G),(U46A)
D31973.1/986-1036	(A1G),(A9C),(C25G),(U30G),(U33A),(C36U),(U46C)
D87358.1/983-1033	(A9G),(A15U),(C25G),(U30C),(U33G),(C36U),(U46A)
D87356.1/983-1033	(A1G),(G2A),(A9G),(A15U),(C25G),(U30C),(U33G),(C36U),(C45U),(U46C)
D84263.2/9267-9317	(A9G),(A17U),(C25G),(U30C),(U33G),(C36U),(A39G),(U46A)
AY973865.1/1663-1713	(A1G),(G2A),(A9G),(A15U),(C25G),(U30C),(G32A),(U33G),(C36U),(C45U),(U46C)
D86543.1/983-1033	(A1G),(A9G),(A15U),(A20G),(C25A),(U30C),(U33C),(C36U)
D87360.1/983-1033	(A1G),(G2A),(A9G),(A15U),(C25G),(U30C),(G32A),(U33G),(C36U),(C45U),(U46C)
AY878650.1/9259-9309	(A9G),(A15U),(C25G),(U30C),(U33G),(C36U),(U46A)
D87354.1/983-1033	(A9G),(A17U),(C25G),(U30C),(U33G),(C36U),(A39G),(U46A)
D49777.1/983-1033	(A1G),(A9C),(C25G),(U30G),(U33A),(C36U)
D84264.2/9276-9326	(A9G),(A15U),(C25G),(U30C),(U33G),(C36U),(U46A)
D87357.1/983-1033	(A9G),(A15U),(C25G),(U30C),(U33G),(C36U),(U46A)
D38079.1/983-1033	(A9G),(A17U),(C25G),(U30C),(G32A),(U33G),(C36U),(A39G),(U46A)
D84398.1/983-1033	(A1G),(A9C),(C25G),(U30G),(U33A),(C36U),(U46C)
AY859526.1/9242-9292	(A1G),(G2A),(A9G),(A15U),(C25G),(U30C),(G32A),(U33G),(C36U),(C45U),(U46C)
D87355.1/983-1033	(A9G),(A15U),(C25G),(U30C),(U33G),(C36U),(U46A)
AY973866.1/1663-1713	(A1G),(G2A),(A9G),(A15U),(C25G),(U30C),(G32A),(U33G),(C36U),(C45U),(U46C)
D37855.1/983-1033	(A1G),(G2A),(A9G),(A15U),(C25G),(U30C),(U33G),(C36U),(C45U),(U46C)
D84262.2/9289-9339	(A1G),(G2A),(A9G),(A15U),(C25G),(U30C),(U33G),(C36U),(C45U),(U46C)
D84265.2/9273-9323	(A1G),(A9G),(A13G),(C25G),(U26C),(U30C),(U33G),(C36U)
D50409.1/9341-9391	(A1G),(A9C),(C25G),(U30G),(U33A),(C36U),(A39G),(U46C)
D87353.1/983-1033	(A1G),(A9G),(A13G),(C25G),(U26C),(U30C),(U33G),(C36U)
D87359.1/983-1033	(A9G),(A15U),(C25G),(U30C),(U33G),(C36U),(U46A)
D87363.1/983-1033	(A1G),(G2A),(A9G),(A15U),(C25G),(U30C),(G32A),(U33G),(C36U),(C45U),(U46C)
D37860.1/983-1033	(A9G),(A17U),(C25G),(U30C),(G32A),(U33G),(C36U),(A39G),(U46A)
D87362.1/983-1033	(A1G),(G2A),(A9G),(A15U),(C25G),(U30C),(G32A),(U33G),(C36U),(C45U),(U46C)

See text for a comparison of this table produced by RNAmutants with the experimental mutagenesis study of You et al. [39].

**Table 3:** Distribution of the mutations inside versus outside the evolutionarily conserved RNA stem loops SLI to SLVII corresponding to the profiles of Figure 12

Frame size	50 nt		100 nt		150 nt	
Location w.r.t. RNA regions	In	Out	In	Out	In	Out
All mutations	48%	52%	39%	61%	38%	62%
In a base pair of size ≥25 nt	41%	59%	27%	73%	24%	76%

The first row presents statistics computed for all mutations, while the second row presents statistics for mutations involved in a base pair (*i*, *j*) of length |*j*−*i*|≥25.

The 33 sequences mutating at index 35 and 36 have also several other significant mutations. Most of these mutations are similar. Assuming that these mutants are viable, this suggests that some of these additional mutations offset the deleterious effect of C35G or C36G. A complete analysis of all these sequences would be too demanding, but we can illustrate this phenomenon by looking at the 3 sequences associated with C35G.

Three mutations (A15U, C25G, and A39G) are found simultaneously in all occurrences of C35U. The mutations C25G and A15U are located at the extremities of the hairpin loop in the native structure, and more specifically, C25G creates a potential base pair with nucleotide U at index 14 (and potentially also with nucleotide U at index 15). We conclude that these two mutations tend to stabilize the upper helix and counterbalance the deleterious effect of C35G. The role of A39G remains more obscure. In [39], You et al. observed that this mutation (A92G in the paper) is lethal. However, structure probing did not reveal any irregularity in the cleavage product of this RNA, suggesting that the sequence of the bulge is affected rather than the global structure. A potential structural use of this mutation would be to prevent the creation of base pairs supporting the disruption of the upper helix through C35G. An analysis using the thermodynamic model with RNAmutants tends to support this hypothesis.

An interesting case is for A11G which occurs, in a single sequence (AF054264.1:326–376) from the Rfam seed alignment, together with A1G. This has been reported as one of the clones used in [44]. From this study, it remains unclear how replication is affected by these mutations; however, the possibility of a deleterious effect of A11G is potentiality (indirectly) supported by this work.

The following group of predicted deleterious mutations involves the nucleotide C at position 29, either directly (C29G) or indirectly through a base pair (C19G, U16G, C12G). If no specific analysis has been performed for this mutation, it appears that C29G can be found in two nonviable mutants (5BSL3.2 mutA and 5BSL3.2 mutB) in [39]. The deleterious nature of C29G has not been validated, but the destabilization effect of this mutation in the upper helix is suggestive.

Other minor mutations which do not disrupt the native structure are identified. With a break number of 0, these mutations cannot be considered as deleterious. However, some of them have been detected to alter replication (C74U and C77U or C21U and C24U in our notation) in [39]. One potential explanation suggested by our results is that the local structure of the hairpin loop is affected, rather than that of the global secondary structure.

### Scan of HIV *trans*-activation response reveals regions under evolutionary pressure

In this section, we show how RNAmutants can be used to detect regions of the sequence which have been optimized during evolution. We restrict mutations to a 3-nucleotide frame and slide the latter on sequences. The frames associated with an alteration of the functional structure in 3-mutants are most likely optimized to preserve the structure, and are thus identify under a *purifying* selective pressure. Our results reveal critical regions in the *trans*-activation response element of the human immunodeficiency virus and suggest applications for RNA drug design.

For this study, we use sequences of human immunodeficiency virus *trans*-activation response (HIV TAR) from the HIV-1 genome. The Rfam seed alignment contains 426 sequences of length 57 nt with an average identity of 91%. This RNA element is critical for the *trans*-activation of the viral protomer and virus replication. The TAR hairpin acts as a binding site for the Tat protein and this interaction stimulates the activity of the long terminal repeat promoter. Previous studies have shown that the 3 nt bulge from index 22 to 24 is essential for binding [36]. Moreover, the 3D structure of the 6 nt apical loop (index 29 to 34) is indispensable for *trans*-activation of the viral protomer and virus replication [37]. This RNA element is a potentially important drug target [38]. Its consensus secondary structure is shown in Figure 6.

We are interested in detecting regions which have been selected during evolution to preserve a specific pattern, for structural or functional purposes. For each sequence in the dataset, we slide an open frame and allow mutations in this region only. Then, we sample structures from this model, and measure the centroid and average distances.

**Figure 8:**
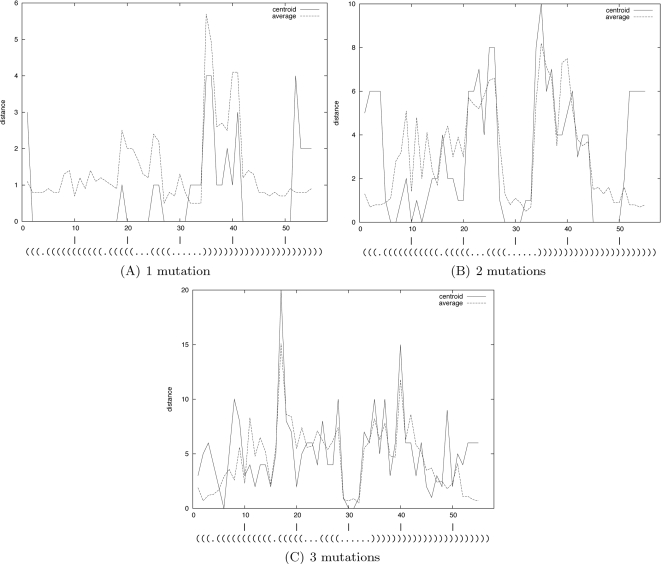
Scan of 57 nt human immunodeficiency virus *trans*-activation response elements (HIV-1 TAR) from the HIV-1 genome. By sliding a window forward, for each 3 nt window in the Rfam seed alignment of HIV-1 TAR elements, we allow mutations only within this window, and subsequently compute the centroid and average distances. The starting position of the 3 nt window is given on the *x*-axis and the centroid (resp. average) distance is given on the *y*-axis. (See Results/Discussion for the definition of centroid and average distance.) Each curve shows the results computed with a fixed number of mutations in the frame: 1 mutation (A), 2 mutations (B), and 3 mutations (C).

Here, the size of the open frame is chosen to fit the length of the bulge (i.e., three nucleotides). Larger frame sizes would result in an attenuation of the signal (data not shown). For each starting position of the frame (1 to 55), we compute the mean *centroid* distance and mean *average distance* for each sequence in the dataset. These curves are displayed in Figure 8. The secondary structure annotation is given at the bottom of each of these three graphs (one for each number of mutations in the open frame).

The secondary structure can be decomposed into four distinct patterns which are: (1) a pairing (denoted S1 for stem 1) between regions (17,21) and (39,43), (2) a bulge at index (22,24), (3) another pairing (denoted S2 for stem 2) between regions (25,28) and (35,38), and (3) a hairpin at index (29,34).

We look first at the curves with a single mutation inside the frame—see Figure 8A. A clear signal appears at index 35–36 and 40–41: Both curves (average and centroid) show a clear peak at these positions. The regions associated with this signal correspond exactly to the 3′-end regions involved in the two stems S1 and S2. We observe a mirror effect when the frame matches the 5′-end regions; two other peaks emerge at index 18 and 25–26. Interestingly, the magnitude of these two peaks is significantly lower than those of the first ones, indicating that the 3′ regions have been potentially under a higher selective pressure.

When two mutations are allowed inside the frame (see Figure 8B), the phenomenon observed above is amplified. The asymmetry between the two paired regions of S2 is almost cancelled, but not for those of S1. In addition, a clear signal now appears when the frame matches the bulge. It may also be interpreted as a signal indicating that this region has been constrained along evolution.

Finally, when three mutations are performed inside the frame (see Figure 8C), the signals mentioned before can still be identified, but tend to be washed out by the noise. Indeed, when all positions in the frame mutate, the sequence is so denatured that the conservation of the secondary structure would require an optimization of the surrounding sequence. This remark is related to the observation given below for the hairpin region.

Additionally, two clear peaks now appear when the frame matches the paired region of the stem S2. This may be a correction of the weakness of the signal observed in the previous graph (Figure 8B). It also confirms that both these regions may have been optimized to base-pair.

For these three graphs, it is remarkable to notice that mutations inside the subsequence of the hairpin never really affect the global structure of the RNA element. It may be suggested that the sequence outside the hairpin has been optimized to prohibit any stable interaction with the central region in order to stabilize the secondary structure and facilitate the formation of the complex 3D motifs observed in [37].

According to these observations, four sequence optimizations may have been performed for these sequences. The first two are for the regions paired to each other through the stem S1 and S2. This may be justified by the need for these sequences to pair to each other in order to stabilize the bulge and the hairpin lying between them. It also appears that the sequence of the bulge cannot tolerate two mutations. Our analysis suggests that evolutionary pressure has selected these nucleotides to facilitate the formation of the bulge required for the binding. Finally, the global structure does not seem to be affected by mutations inside the hairpin loop. As it has been said before, this suggests an optimization of the surrounding sequence to stabilize this loop and allow the formation of a complex 3D motif inside the apical loop.

These results suggest that a method combining RNA binding predictors [45],[46] and secondary structure prediction software [14],[15] with RNAmutants could be a successful and promising approach for the prediction and design of functional RNAs.

Scan of the 3′ UTR of GB virus C reveals how evolution shaped the sequence. We conclude the results section with a series of computational experiments on the 3′ UTR of the GB virus C (GBV-C). By scanning this RNA sequence, we show how RNAmutants can provide evidence that different regions have been optimized to conserve RNA secondary structure even in the presence of pointwise mutations. In particular, we show that the sequence has been designed to prevent deleterious effects of mutations on the evolutionarily conserved stem-loops. This work suggests potential large-scale applications of RNAmutants for genome-wide scanning purposes.

In recent years the structure of RNA viruses in the family of *Flaviviridae* has received particular attention [47]. Here, we focus on the 3′ UTR of the Hepatitis G virus (GB virus), a single-stranded positive-strand RNA virus with GenBank/EMBL accession number AB013500, whose secondary structure has been determined using both thermodynamics and evolutionarily information [48]. This 311 nt sequence has the advantage of containing a balanced number of nucleotides located *within* regions having an evolutionarily conserved secondary structure (167 nt), as well as *outside* of any region having conserved secondary structure (144 nt). The conserved secondary structure is composed of seven stem-loops numbered from SLI to SLVII.

We aim to study how evolution shaped this sequence, and to provide some evidence that certain regions have been thermodynamically optimized. In a manner similar to that of Vienna Package program RNAplfold [49], we scanned the 3′ UTR GBV-C RNA sequence with a moving window of fixed size, and analyzed the distribution of mutations and base pairs in *k*-mutant ensembles of each window.

Sliding a window of size *L* over this sequence, we extracted 311−*L*+1 subsequences and ran RNAmutants to sample mutated sequences and their secondary structures. Here, we use the following notation. Let *ω* denote the complete sequence of the 3′ UTR of GBV-C (length *N* = 311), and let *W_i_^L^* denote the subsequence of size *L* starting at index *i*. Let *SW_i_^L^*(*k*,*n_s_*) denote the set of *n_s_* many *k*-mutant sequences and secondary structures computed from *W_i_^L^*. The full set of sequences scanned by RNAmutants is denoted by 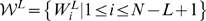, and the sample set of *n_s_ k*-mutants and structures computed from 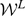 is denoted 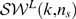.

The *probability* of a base pair (*i*, *j*) in 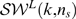 is defined as the number of occurrences of (*i*, *j*) in the secondary structure samples divided by the number of samples computed for a sequence that can potentially form a base pair between indices *i* and *j* (e.g., *W_k_^L^* such that *j*−*i*<*L*). Formally
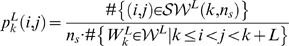
(13)This measure, motivated by that from the Vienna Package program RNAplfold [49], averages the frequency of occurrence of base pair (*i*, *j*) in the ensemble of *k*-point mutants, over all size *L* windows containing both *i*, *j*.

For this set of computational experiments we chose a frame size *L* = 50 and chose the number *n_s_* of sampled *k*-mutant sequences and structures to be 1000, for each *k* from 0 to 8. These values were chosen to provide a good balance between the computation speed (a bounded, yet somewhat deep search in mutation depth) and maximal range *j*−*i*<*L* of base pair (*i*, *j*). For comparison, the default value for window size used in RNAplfold is 70.

**Figure 9:**
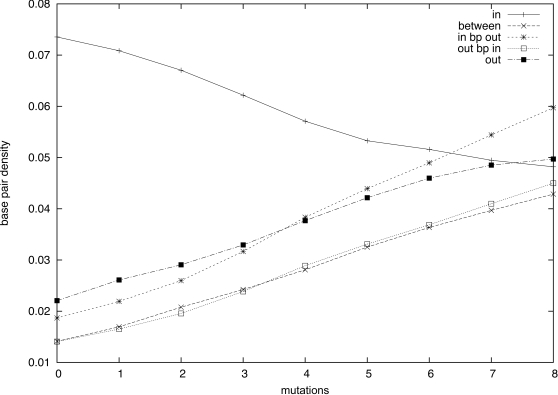
Base pair density in *k*-mutants (0≤*k*≤8). The *x*-axis represents the number of mutations, while the *y*-axis represents the (normalized) frequency of base pairs (*i*, *j*) (*i*<*j*) over all windows containing both extremities *i*, *j*. Results are classified into five different instances: (1) *i* and *j* belong to the same stem region, (2) *i* and *j* are in two different stem regions, (3) *i* is in a stem region and *j* is not, (4) *j* is in a stem region and *j* is not, and (5) neither *i* nor *j* belong to any stem region.

The first analysis aims to estimate the *density* of base pairs in the different regions—regions of evolutionarily conserved stems, denoted by *stem region* or *inside region*, and regions having no evolutionarily conserved stems, denoted by *non-stem region* or *outside region*. We clustered the base pair density values in five cases according to the location of each index *i*, *j* of base pair (*i*, *j*): (1) *i* and *j* are two indices belonging to the same stem region, (2) *i* and *j* are in two different stem regions, (3) *i* is in a stem region and *j* in a non-stem region, (4) *i* is in a stem region and *j* is in a nonstem region, and (5) *i* and *j* do not belong to any stem region. Then, we plotted these base pair density values with respect to the number of mutations in samples. The results computed with the parameters given above (*L*  =  50, *n*_s_  =  100, and 0≤*k*≤8) are shown in Figure 9. Note that the count done in the denominator of Equation 13 respects the same classification constraints and ensures normalization of the estimator values.

The figure shows very distinct behavior for base pairs occurring inside the same stem region (1) versus other possibilities (2–5). As expected when no mutation is allowed (i.e., *k* = 0), he base pair density appears to be higher for base pairs in stem regions. This means that these regions are more structured than the others. (This argument does not suggest that nonstem regions are not structured but only that they are locally less optimized.) However, when the number of mutations increases, all curves tend to reach an equilibrium, with approximately equal density for each of the five cases. While density for base pairs in the same stem, case 1, decreases with an increasing number of mutations, density for the other four cases increases. This phenomenon suggests that selective pressure has been applied to ensure robustness of (local) structure in the 3′ UTR GBV-C RNA with respect to mutation. Putatively, an inflection in the curve of stem regions appears at roughly 4 mutations in the figure. This remark will take its importance later in the discussion.

**Figure 10:**
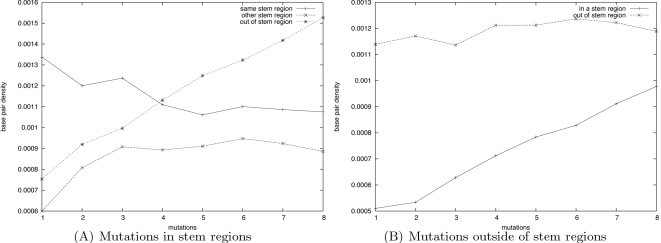
Relative propensity of mutations occurring inside (A) and outside (B) of stem regions to base pair inside or outside the same region. The statistics have been computed using a scanning window of size 50 with up to 8 mutations. When a mutation occurs in a stem region 10(A), we distinguish three cases: when the base pair is created inside the same stem region, when the base pair links another stem region and when the mutation base pairs outside any stem regions. In the case of a mutation happening outside the stem regions 10(B), we only need to distinguish whether the base pair links a stem region or not.

The next study aims to analyze the base pairing preferences of mutations regarding their location in the sequence. Using the same set of computational experiments, we investigated the distribution of base pairs (*i*, *j*) involving a mutation at one of their extremities (i.e., index *i* or *j* mutates). We computed the base pair probability *m_k_^L^*(*i*,*j*) restricted to these specific base pairs and normalized the results (i.e., we divided the base pair density by the number of mutations allowed in the sample set)
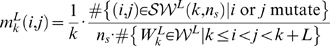
(14)Then, we clustered the results according to the same classification of base pairs as above and computed the base pair density in each cluster. Results are shown in Figure 10. For clarity of discussion, in the left panel of this figure we plotted the curves associated with a mutation occurring in the stem region (Figure 10A), while the right panel displays the curves associated with a mutation occurring outside the stem region (Figure 10B).

Figure 10A reveals that in the close neighborhood (small number of mutations) of the wild sequence, the mutations occurring in a stem region base-pair preferentially inside the same stem region. An increase in the number of mutations has very different consequences on the density of base pairs in the different clusters. In agreement with our previous observations, the number of mutations created inside the same stem region decreases. In contrast, if the densities increase in the two other cases, we observe a clear preference for creating a base pair outside any other stem region. Indeed, while the behavior of the two curves (base-pairing in another distinct stem region, and outside) have similar behavior for small number of mutations, it turns out that roughly beyond 4 mutations, more mutations tend to base-pair outside and “protect” as much as possible the cleavage between the stem regions.

Symmetrically, when few mutations are performed outside the stem regions (cf. Figure 10B), we observe a clear preference for base pairings in the same region, thus preserving the stems from destabilization by mutations occurring in the nonstem regions. However, in agreement with previous observations, larger numbers of mutations tend to progressively equilibrate the distributions by increasing the base pair density of mutations base pairing in stem regions. This observation suggests that non-stem regions have been constrained to prevent mutations from interacting with stems to disrupt the structure.

**Figure 11:**
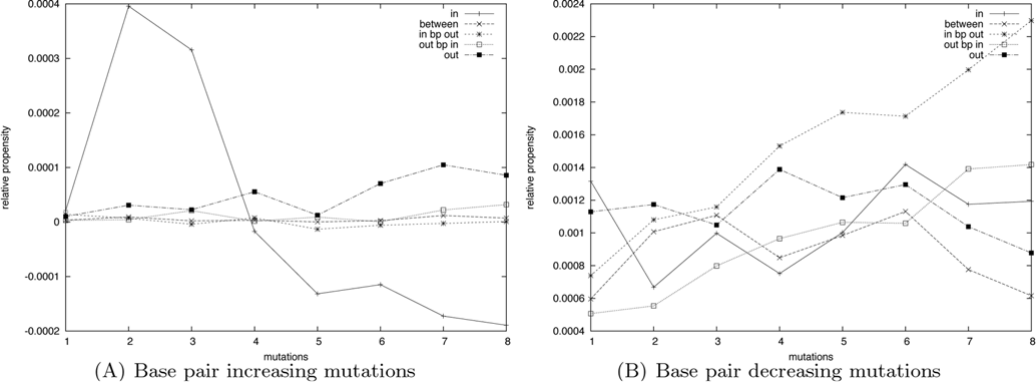
Differential probability of mutation associated with a base pair increasing mutation (A) or a base pair decreasing mutation (B). The *x*-axis represents the number *k* of mutations, while the *y*-axis represents the differential probability *p^k^* ^+*1*^ (*i*, *j*)−*p^k^* (*i*, *j*).

We now investigate the distribution of mutations that increase the base pairing probability (called *base pair increasing mutations*), versus those that decrease base pairing probability (called *base pair decreasing mutations*). To evaluate the evolution of these probabilities from one level of mutation *k* to the next *k*+1, we compare the local base pairing probabilities *p^k^* (*i*, *j*) computed from 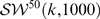 (e.g., sample set with *k* mutations) with those computed from 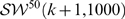. Then, we estimate the difference *p^k^* ^+1^ (*i*, *j*)−*p^k^* (*i*, *j*), subsequently called the *differential probability*. We show the corresponding curves in Figure 11, where the results have been once again classified into five clusters.

The distribution of base pair increasing mutations (cf. Figure 11A) presents some interesting features. Indeed, when a single mutation is performed, we first observe a tendency to stabilize the structures already existing in and out the stem regions, thus conserving the existing structure of the full 3′ UTR GBV-C RNA sequence. However, afterward, an increased number of mutations tends to be more favorable to mutations strengthening the base pairs between a stem and a non-stem region. Simultaneously the probability of mutations favoring base pairs inside stem regions increases to a lesser extent. Interestingly, if the probability of base pair increasing mutations for bases occurring between two distinct stem regions seems also to increase for small values of *k*, it turns out that these probabilities tend to remain identical afterwards (e.g., the differential values decrease).

The case of base pair decreasing mutations is in fact much more interesting since essentially only base pairs inside stem regions seem significantly affected by such mutations, although single mutations appear not to have any significant effect (differential probability close to zero). The two next levels (*K* = 2, 3) present a remarkable peak which completely collapses for a further increasing number of mutations (*k*≥4). The negative values indicate that the probability of base pair decreasing mutations inside the stem are decreasing, and thus that stabilization occurs once a few mutations have been occurred to locally reorganize the structure. This clear signal could prove useful in detecting structured regions of a genome, and possibly help identify subsequences under evolutionary pressure. Interestingly, the change of sign of the differential base pairing probability in the same stem region happens for 4 mutations, which correlates with the putative inflection point in Figure 9 for the base pair density curve for the same cluster of base pairs.

Finally, we study the distribution of mutations in the complete 3′ UTR GBV-C RNA sequence. In complement to the previous experiments performed with a frame size of 50 nucleotides and thus restricted to local considerations, we now also provide an insight on the influence of mutations, sampled from the Boltzmann *k*-point mutant ensemble, on the medium and long range base pairing by including statistics computed with larger frame sizes. Using the equation 14, we estimate the mutation base pair probability in the sample set and derive the *average mutation probability* from these values.

**Figure 12:**
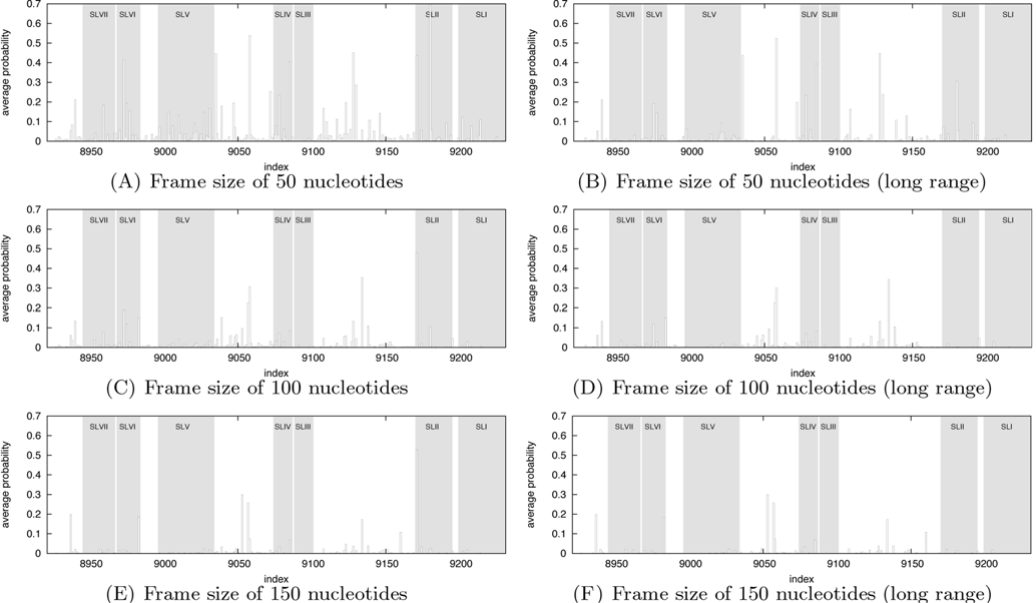
Average mutation rates computed from a scan of the 3′ UTR of GB virus C (GBV-C) with frames of 50 (A and B), 100 (C and D), and 150 nucleotides (E and F). Evolutionarily conserved stem loops identified in [48] are indicated with shaded regions. Profiles with no restriction on the length *j*−*i* of the base pair (*i*, *j*) associated with the mutations are given in the left column, while those for medium and long range base pairing (length ≥25 nt) are shown in the right column.

The *average mutation probability* at index *i* in a sample set of *k*-point mutants is defined as the sum of the mutation base pair probabilities *m_k_^L^*(*i*,*j*) (i.e., *m_k_^L^*(*i*) = Σ*_j_ m_k_^L^*(*i*,*j*)). Additionally, in order to investigate the influence of medium and long range base pairing on the mutation distribution, we also computed the values of *m_k_^L^*(*i*) restricted to base pairs (*i*, *j*) with |*j*−*i*|≥25. Mutation profiles computed using this procedure are given in Figure 12.

The distribution of the mutations inside and outside stem regions is evaluated as the sum of the mutation probabilities *m_k_^L^*(*i*) in both regions normalized by the number of nucleotides in these regions (166 in stem regions and 144 outside). The numerical results given in Table 3 summarize these statistics for the general case as well as the case of mutations involved in a medium to long range base pair, i.e., base pairs (*i*, *j*) whose extremities *i*, *j* are at a distance of at least 25 nucleotides. Average mutation rates for such medium to long range base pairs are depicted in Figure 12.

**Figure 13:**
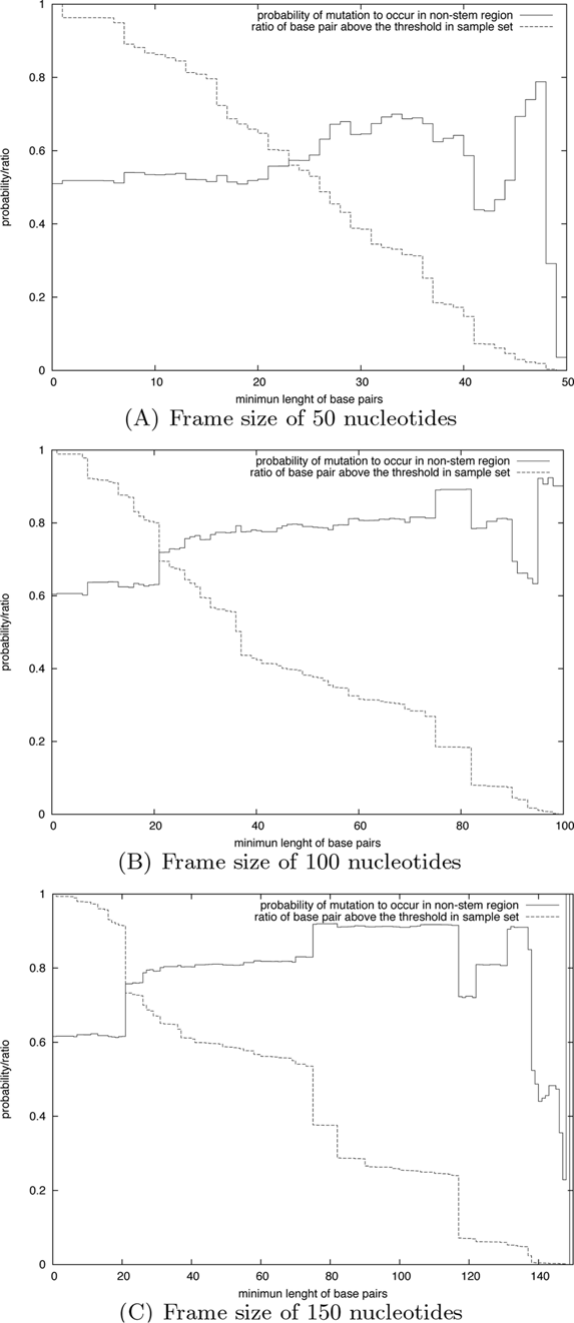
Probability of mutations occurring in a base pair (*i*, *j*), whose length *j−i* exceeds a certain threshold. The *x*-axis represents the threshold value for base pair length. Results are reported for frame sizes of 50 (A), 100 (B), and 150 (C). The fractions of mutations satisfying the criteria in the sample set are given using the dashed line.

Since the threshold used to filter short range base pairs may seem arbitrary, for the sake of clarity of discussion, we include graphs representing the values obtained for all possible threshold values together with the ratio of samples satisfying the cut-off in the sample set. Figure 13 illustrates these statistics. The *x*-axis represents the minimal base pair length while the *y*-coordinates give the fraction of mutations in non-stem regions (plain line) and the fraction of samples satisfying the threshold (dashed line).

In this study, we used frame sizes of *L*  =  50, 100, and 150 nucleotides and computed 1,000 samples with *k*  =  1 mutation (results with 2 mutations were also computed and produced the same results). Frame sizes larger than 150 nucleotides have not been considered since only few base pairs distanced at more than 150 nt appeared in our sample sets. (See Figure 13. As shown in the supplementary Figure 1, the RNAfold dotplots of the full sequence confirmed this observation.)

The distribution of mutations between structured (stem regions) and nonstructured regions presents a small but significant bias in the general case. When the requirement on the minimal length of base pairs is applied, this signal is strong and surprisingly clear. This observation suggests that in the fitness model [29], [50]–[52], evolution has constrained medium and long range base pairing to favor mutations outside evolutionarily conserved stem regions. This remark automatically suggests the potential usefulness of RNAmutants in gene discovery based on clustering of RNAmutants statistics. This hypothesis is the subject of current research on larger scale studies.

## Supporting Information

Figure S1 RNAfold (a) and RNAplfold (b) Dotplots of the 3′ UTR GB Virus C. Stem regions are annotated with red boxes.
(0.10 MB PDF)
