# Supplementary figures and images for: Efficient Algorithms for Probing the RNA Mutation Landscape
Source: PLoS Comput Biol. 2008 Aug 8;4(8):e1000124. doi: 10.1371/journal.pcbi.1000124 (PMC2475669; doi:10.1371/journal.pcbi.1000124)

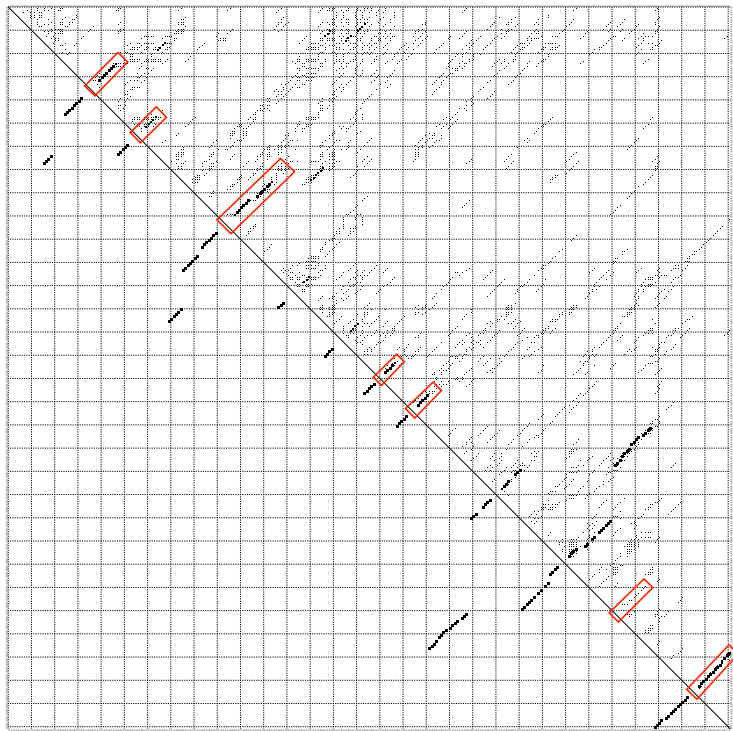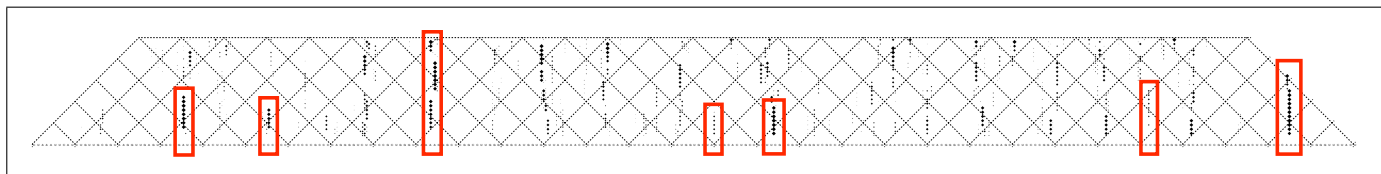

Supplement: Figure S1 — RNAfold (a) and RNAplfold (b) Dotplots of the 3′ UTR GB Virus C. Stem regions are annotated with red boxes. (0.10 MB PDF) [file pcbi.1000124.s001.pdf]
